# State-of-the-Art Trends in Data Compression: COMPROMISE Case Study

**DOI:** 10.3390/e26121032

**Published:** 2024-11-29

**Authors:** David Podgorelec, Damjan Strnad, Ivana Kolingerová, Borut Žalik

**Affiliations:** 1 Faculty of Electrical Engineering and Computer Science, University of Maribor, Koroška cesta 46, SI-2000 Maribor, Slovenia; damjan.strnad@um.si (D.S.); borut.zalik@um.si (B.Ž.); 2 Department of Computer Science and Engineering, University of West Bohemia, Technická 8, 306 14 Plzen, Czech Republic; kolinger@kiv.zcu.cz

**Keywords:** data compression, data restoration, universal algorithm, feature, residual

## Abstract

After a boom that coincided with the advent of the internet, digital cameras, digital video and audio storage and playback devices, the research on data compression has rested on its laurels for a quarter of a century. Domain-dependent lossy algorithms of the time, such as JPEG, AVC, MP3 and others, achieved remarkable compression ratios and encoding and decoding speeds with acceptable data quality, which has kept them in common use to this day. However, recent computing paradigms such as cloud computing, edge computing, the Internet of Things (IoT), and digital preservation have gradually posed new challenges, and, as a consequence, development trends in data compression are focusing on concepts that were not previously in the spotlight. In this article, we try to critically evaluate the most prominent of these trends and to explore their parallels, complementarities, and differences. Digital data restoration mimics the human ability to omit memorising information that is satisfactorily retrievable from the context. Feature-based data compression introduces a two-level data representation with higher-level semantic features and with residuals that correct the feature-restored (predicted) data. The integration of the advantages of individual domain-specific data compression methods into a general approach is also challenging. To the best of our knowledge, a method that addresses all these trends does not exist yet. Our methodology, COMPROMISE, has been developed exactly to make as many solutions to these challenges as possible inter-operable. It incorporates features and digital restoration. Furthermore, it is largely domain-independent (general), asymmetric, and universal. The latter refers to the ability to compress data in a common framework in a lossy, lossless, and near-lossless mode. COMPROMISE may also be considered an umbrella that links many existing domain-dependent and independent methods, supports hybrid lossless–lossy techniques, and encourages the development of new data compression algorithms.

## 1. Introduction

Data compression is the process of converting an input data stream (the source stream or the original raw data) into another data stream (the output or the compressed stream) that has a smaller size [1]. It represents one of the oldest and most traditional disciplines of computer science.

Data compression started with the statistical methods of Shannon [2] and Huffman [3]. Shannon introduced the concept of information entropy H(X) for a random variable *X*, which is computed by (1).

(1) $$H\left(X\right)=-\sum_{i=1}^{\left|\Sigma \right|}p_{i}\log_{2}\left(p_{i}\right)$$

Here, *X* takes values (symbols) from the alphabet Σ, and $p_{i}$ is the probability that the symbol assigned to *X* is $\sigma_{i}\in \Sigma$. H(X) in (1) measures the average amount of information (the information content) obtained by selecting one random symbol from Σ with a probability distribution $p_{i},1\leq i\leq \left|\Sigma \right|$. Base 2 of the logarithm specifies that the measurement unit used is a bit. Now, assume that *S* represents the input data stream of symbols that we want to compress into the output stream *Y*, where the symbols in *S* are random variables $\langle X_{1},\ldots ,X_{N}\rangle$ that are independent of each other and have the same probability distributions (we shall thus use a common label *X* for all) and hence the same entropies H(X). These assumptions hold for classical statistical data compression algorithms that treat individual symbols separately, such as the aforementioned Huffman coding. Shannon established the source coding theorem in [2], which informally states that *S* under the above conditions can be compressed losslessly into |Y|≥N·H(X) bits, as N→∞; but conversely, |Y|<N·H(X) bits implies under the same conditions that some information will be lost. However, in practice, we never compress streams of infinite length. The requirement of N→∞ can be omitted if the probability distribution of the alphabet symbols is based on the actual frequencies of the individual symbols in the stream *S*. However, this implies that the alphabet and the probability distribution tailored to the input stream must be transmitted to the decoder together with the encoded stream *Y*. Especially for shorter streams, the contribution of this part is not negligible and significantly degrades the compression rate. An elegant alternative is the adaptive statistical coding [4], where both the encoder and the decoder build the alphabet and update the probability distribution at runtime.

The idea of statistical coding is to assign shorter codes to symbols with higher probabilities. The most advanced and efficient of these methods is arithmetic coding [5], which allows highly probable symbols to be represented with fewer than one bit each. It follows the paradigm that a value from any range of numbers can be assigned to each stream unambiguously.

The lowest boundary of the output stream size |Y| can be beaten by assigning individual codes to longer strings of symbols. Run length encoding (RLE) encodes sequences of identical symbols by the symbol plus the number of repetitions, while dictionary-based compression assigns indices to parsed substrings of generally different symbols and stores each substring into a so-called dictionary (a list or a hierachical search structure). In the 1970s, Ziv and Lempel [6] introduced the first such approach, based on a dynamic dictionary built upon the recently parsed part of the stream. Their LZ77 algorithm was later improved with many variants, the most famous being the LZW [7]. In 2000, Moffat and Stuiver [8] introduced interpolative coding, which can also beat the entropy-based lowest length. It converts a stream into an ascending sequence of integers that it divides recursively during the encoding. Žalik et al. presented an improvement in [9]. All the aforementioned approaches compress data losslessly, which means that *X* and the decoded *Y* are exactly the same. Another possibility to achieve |Y|<N·H(X) is pre-processing with entropy reduction methods such as Burrows–Wheeler [10,11] and Move-To-Front transform [12]. From the latter, the Move with Interleaving (MwI) transform [13] has recently been derived, which is also efficient for data represented with larger alphabets such as greyscale images and audio. Lossless compression algorithms for these types of data are typically based on predictions. Examples include PNG [14] and JPEG-LS [15] for images, and FLAC [1], MPEG ALS [16] and Monkey’s Audio [17] for audio. Note that the use of entropy reduction transformations and predictions changes the distribution of the alphabet symbols. At the same time, the nature of multimedia data is such that it makes sense to take into account the proximity (similarity) of neighboring samples. The residuals obtained as the differences between the predictions and the original values often have a geometrical (Laplace) distribution, which is why, e.g., FLAC and JPEG-LS use variable-length Golomb–Rice coding [1,18,19]. In certain contexts, the aforementioned interpolative coding can also be used. On the other hand, PNG uses DEFLATE [1,20], a combination of the traditional Huffman statistical coding and LZ77 dictionary coding. Specifically, the color indices frequently used in PNG are not geometrically distributed, and the filters (predictions) used produce a lot of repeating patterns in both the indexed and the True Color mode. Nevertheless, multimedia data, including images, audio and video, are nowadays mostly compressed by lossy algorithms.

The ubiquity and size of multimedia data has made it clear that before compression, it is necessary to cut into the data radically and discard (a large) part of them irretrievably, without making the losses visible or at least not disruptive to the user. The solution for efficient lossy compression was found in signal theory, where the Fourier transform and later the wavelet transform were the main tools for determining the frequency content of a signal. In digital audio, frequencies computed with a discrete Fourier transform are grouped into subbands, and these are further processed by utilizing the psychoacoustic models, aiming to improve the compression on the basis of previously discovered human hearing characteristics (frequency masking, logarithmic pitch perception, frequency-dependent loudness curves) [21]. In the image domain, researchers have, facing the market demands, also turned to frequency content analysis. JPEG, the first standard for lossy compression of raster images with continuous color tones, is based on the Discrete Cosine Transform (DCT) [22]. However, since the DCT has time complexity $O(n^{3})$, the image must be split into blocks of 8×8 pixels, which limits the compression efficiency and leads to a blocky image appearance at higher compression ratios. In audio compression, the problem was solved by using the modified DCT (MDCT), where an overlapping between the subsequent blocks is applied. In the image domain, however, the problems were largely solved by replacing DCT with the wavelet transform, operating in linear time complexity O(n) [23]. Consequently, ordinary-size images do not need to be divided into small blocks. JPEG 2000 proved to be very effective with the Daubechies wavelet [24]. The transformation-based data compression methods are still used widely in the mentioned and other domains, even though significant improvements were not achieved in the last two decades.

The above conclusion can be extended to data compression in general. Digital transformation and accelerated digitalization, where new devices collect ever-increasing amounts of highly heterogeneous data, pose greater challenges than ever before. In these circumstances, the data compression has not lost its relevance, but neither has it made significant progress since the beginning of the millennium. The goals of this feature paper are to systematically identify contemporary challenges and the responses of the research community so far, to highlight the problems and, where possible, to propose solutions. Its main contributions are:

- An overview of the challenges driving the recent progress in data compression.
- A critical overview of trends in data compression and how they relate to specific challenges.
- Possibly the first proposal for a formal definition of near-lossless compression, which was occasionally addressed but not clearly specified in the literature in different contexts in the past.
- The introduction of the COMPROMISE paradigm and, within it, the following innovations:
  - Generality, which we achieved by a unified taxonomy of (generic) features to which their domain-specific descriptions can be linked.
  - Universality in the form of a uniform concept to allow lossy, lossless, and near-lossless compression within a common framework.
  - The use of interpolation of intermediate values and not only an extrapolation of upcoming values. Dynamic programming and artificial intelligence techniques can be used as an alternative to greedy methods to optimize predictions. Another advantage of feature-based prediction is that the interpolation parameters are stored losslessly in the feature description, which prevents the accumulation of decoding errors. In the case of extrapolation, a lossy decoded sample is an input to the prediction of the next sample, which obviously means that errors accumulate.
  - Presenting information in a way that is more suitable for human interpretation or machine processing. Access to semantic features is often easier from a compressed stream than from raw data, which significantly supports reusability.
  - Finally, the most innovative outcome is certainly the design that integrates all these innovative partial solutions into a single common methodology. That is, all the trends identified in the paper are highly interdependent and require a holistic approach to integrate them into new paradigms. To the best of our knowledge, a data compression method with such an ambitious set of objectives does not yet exist. Feature-based compression and domain independence seem particularly incompatible.

In line with these contributions, the paper is organized into four sections. Section 2 comments on the challenges driving the development of data compression today, lists the trends in the development of the field, and discusses their relations to individual challenges. In Section 3, we describe our own COMPROMISE methodology and discuss how it follows individual trends. In Section 4, we summarize our findings again and suggest guidelines for future work.

## 2. Actual Trends in Data Compression

Fast growth in the amount of data, which is due to new capturing technologies, improved data quality and accuracy, and new domains, is increasing requirements for storage space (with respect to both the storage capacity and the physical area of data warehouses) and higher speeds of data transfer. According to the analysis of the European Commission from 2018, the expected global increase in data by 2025 is 430%, or from 33 ZB to 175 ZB [25]. This is reflected in the energy footprint, due to the operation of server systems and the cooling of computer equipment and rooms, which is also pointed out by the European Initiative for Energy and Environmental Efficiency in the ICT sector [26].

In 2018, the European Commission estimated that the value of data in the European Union will grow from EUR 301 billion to EUR 829 billion in 2025 [25]. However, the data have value only if we know how to use them and if they are accessible. Modern telecommunication networks allow higher speeds of data transfer, but the number of users and their data access demands are growing even faster. In the epidemic situation at the beginning of the current decade, when education and work were being carried out online, network operators reported a significant increase in internet traffic (close to 50% globally). However, even after the pandemic stopped, the situation did not return to its original state.

Important sources of data today are sensors and measuring devices of the Internet of Things (IoT). These devices are designed for ubiquitous mass use, so they need to be affordable and are consequently subject to resource constraints including limited CPU, memory, and power. In line with the edge computing paradigm, these edge nodes are capable of making certain decisions based on the local processing of the captured data, while at the same time, they forward most of the data over the network to other nodes for further processing and archiving. Data compression in the edge nodes must thus be adapted to these constraints. It should not significantly slow down local processing, which usually has to be performed in real time, and the forwarding of data to the network should also be performed with low latency. At the same time, it must be energy-efficient, as edge nodes are often battery-powered, so any computationally demanding compression algorithms are out of the question. Besides this, a node can also collect heterogeneous data, and its memory capacity does not allow the storage of a large number of domain-dependent compression methods, so it is desirable to have an algorithm that is as general as possible. The devices may vary in performance, so scalability is also an expected feature. Finally, further processing at distinct nodes (usually supercomputers) often requires the most accurate data possible, so lossless compression algorithms are often used, which, while not performing computationally demanding transformations, do transmit much larger amounts of data to the network. As a consequence, a compressive sensing paradigm has evolved, which we describe in Section 2.3.

Another important source of large-scale datasets, where compression plays a key role, is high-performance computing (HPC). These datasets are generated by various simulators of natural phenomena, such as physical models of the solar system, weather, or vegetation growth, often as new layers upon the IoT data received from numerous, often heterogeneous edge nodes (e.g., remote sensing data can be used to build a digital terrain model, and information on scanned organs or DNA can be extracted from medical data). The scientific simulations generate and use highly sensitive data, which requires lossless or lossy data compression with a user-controlled error rate. Data compression and reconstruction should not significantly slow down the data processing performance, and therefore, computationally efficient algorithms with low latency should be provided. They should also be designed for parallel processing and different hardware architectures, provide scalability, and handle different types of data, as scientific data processed on HPC supercomputers are typically 1D, 2D, 3D or 4D floating point numbers or integers.

### 2.1. Feature-Based Data Compression

A feature is a piece of information that possesses high discriminative/predictive value for the human interpretation or machine processing (e.g., computer vision, classification) of a data stream.

Although this is a seemingly simple informal definition, it can be interpreted in two ways. Feature can be an attribute or dimension in a feature vector, as we are used to in machine learning. Alternatively, a feature can denote a pattern of basic data elements (samples), e.g., a segment in a segmentation, which is closer to the definition of shape features in feature-based design in geometric modeling. It is usually expected that the samples in a feature share some common property, which can be represented more compactly in comparison to the list of the incorporated sample values. In this paper and in the proposed COMPROMISE methodology described in Section 3, we use this second interpretation to characterize the features.

In the domain of raster images, several geometric features were proposed [27]. Generally, Voronoi diagrams and their dual structures Delaunay simplices are a useful tool. One of the more successful simplex-based methods uses data-dependent triangulation [28]. The result is a sparse matrix, which can be encoded using various strategies. The reconstruction procedure is completely different. The sparse matrix is used to construct Delaunay triangulation [29], over which the color interpolation is performed. Besides triangles, the typical geometric features are isolated points, edges, and symmetries. Chain codes [30,31,32] can also be used for the compact description of objects in images. Compared with the basic raster image representation, chain codes reduce both the number of data, as well as their entropy, thereby allowing lossless compression of an object’s contour using approximately 1 bit per pixel. Another application of chain codes is to describe region borders (features) in segmented images, as we showed in [33]. Regions themselves are filled using stored colors or functional features, such as gradients, during restoration. Features are also employed for data compression in other domains. Even the patterns used in traditional dictionary-based string compression methods (LZ77, LZW) can be called features. In audio compression, features are reference curves that approximate the audio signal [1]. In biomedical signal compression, features depend on the signal modality (ECG, EEG, or other), but, generally, they are wavelets approximating the original signal [34]. A multichannel biomedical signal compression technique was also proposed recently, which considers each channel as a single feature [35]. Chain codes [36,37] are used for compact representation of structures that generate sparse voxel grids.

An important advantage of using features is their reusability. As we have stressed already, raw data are typically used to produce higher semantic data layers which are in fact features, meaning that they do not have to be detected from the raw data every time they are used. This can significantly speed up data processing and also have a positive impact on energy consumption.

Features have great potential for compression, especially in combination with predictions. The idea of a prediction is to derive the observed values from other values in a data stream and in this way produce a new stream of residuals with lower entropy, which allows more efficient compression. A residual is the difference between the original (raw data) and predicted sample value. Particularly for multimedia data, where the continuous nature of signals leads to the Laplace distribution of differences of successive samples (the simplest predictor), predictions are the main tool for lossless compression. However, to our knowledge, the use of features in data compression is entirely domain-dependent, with only a single type of feature typically available per application. Our methodology proposed in Section 3 thus has additional relevance as it can use different types of features arranged into a unified taxonomy of (generic) features, to which the descriptions of their domain-specific interpretation can be linked.

### 2.2. Unified Concept of Lossless, Near-Lossless and Lossy Compression

The idea of combining the advantages of lossless and lossy compression, i.e., the reconstruction quality and the high compression ratio, in a common framework is not new. JPEG 2000 came closest to this by using the integer wavelet transform in lossless mode. In certain cases, the lossless JPEG 2000 can reach a compression ratio of 1:50, but the average efficiency is around 1:3, which is comparable to PNG and JPEG-LS. Of course, JPEG 2000 is a domain-specific algorithm. The bridge between lossy and lossless algorithms could be established with near-lossless compression. In terms of reconstruction capability, this is lossy compression, although near-lossless compression techniques are often derived from lossless ones, where we allow, or even intentionally insert, locally limited errors by modifying or omitting individual data. For example, quiet parts of audio can be replaced with absolute silence. In text compression, we may replace lowercase letters with uppercase, which reduces the alphabet significantly. We can restore such text almost losslessly with a grammar-aware decoder. Nunes et al. [31] introduced the Multiple Grid Chain Code (MGCC) technique for the near-lossless compression of chain codes. They divided the raster image into a grid of 3×3 pixel cells and coded each cell along the raster curve with a code that specifies the pixel pair where the curve entered and left the cell. In collaboration with our Chinese partners [32], we also presented a near-lossless compression of chain codes, where we omitted those pixels in the polygon border chain that were actually in the polygon interior (such a pixel is at the top of a polygon’s concave angle).

In feature-based data compression, lossless entropy coding can be used in all three data compression modes. This means that all errors, typical for lossy and near-lossless mode, are produced in steps before the actual compression. Data restoration (described later in Section 2.3) is an evident source of errors, but not the only one. The quality of the decoded data can easily be manipulated, e.g., by the quantization of residuals or even by omitting some of them (and then interpolating them from the remaining ones).

Let us conclude this subsection with some basic mathematical background for the above descriptions of the three data compression modes. Let *I* and $I^{\prime }$ represent the encoder’s input and the decoder’s output data stream corresponding to a complete regular 3D grid as given in (2) and (3), respectively. Here, $s_{i,j,k}$ and $s_{i,j,k}^{\prime }$ are the values of the samples at the location (i,j,k) in *I* and $I^{\prime }$, respectively, while resX, resY, and resZ represent resolutions (the number of samples) in 3D Cartesian coordinate directions. In the 1D domain, where resY=resZ=1, we may omit indices *j* and *k* (both 0 all the time). Similarly, index k=0 may be omitted in the 2D domain, where resZ=1.

(2) $$I\leftarrow \langle s_{i,j,k}\rangle ,0\leq i<ResX,0\leq j<ResY,0\leq k<ResZ$$

(3) $$I^{\prime }\leftarrow \langle s_{i,j,k}^{\prime }\rangle ,0\leq i<ResX,0\leq j<ResY,0\leq k<ResZ$$

In the lossless data compression mode, $I^{\prime }=I$, which is also described in an expanded form by (4).

(4) $$s_{i,j,k}^{\prime }=s_{i,j,k},0\leq i<ResX,0\leq j<ResY,0\leq k<ResZ$$

In the near-lossless mode, the error is locally controlled as shown in (5), where ε is a user-defined local error threshold.

(5) $$|s_{i,j,k}-s_{i,j,k}^{\prime }|<\varepsilon ,0\leq i<ResX,0\leq j<ResY,0\leq k<ResZ$$

In the lossy mode, the error is globally controlled as shown in (6), where ε is a user-defined cumulative error threshold, and n=resX·resY·resZ.

(6) $$\frac{1}{n}\sum_{i=0}^{ResX-1}\sum_{j=0}^{ResY-1}\sum_{k=0}^{ResZ-1}\left|s_{i,j,k}-s_{i,j,k}^{\prime }\right|<\varepsilon$$

In the case of samples with multiple attributes, such as RGB color components or a pair of stereo audio samples, the difference $|s_{i,j,k}-s_{i,j,k}^{\prime }|$ in (5) and (6) can be computed as the Euclidean distance (7), when all *c* components have nearly the same mean values *E* and nearly the same standard deviations SD in both *I* and $I^{\prime }$. Otherwise, the weighted Euclidean distance (8) may be used, where $w_{h}$ is the inverse variance $\frac{1}{SD_{h}^{2}}$ of the *h*-th component of the samples in both *I* and $I^{\prime }$.

(7) $$|s_{i,j,k}-s_{i,j,k}^{\prime }|=\sqrt{\sum_{h=0}^{c-1}{{(}^{\left(h\right)}s_{i,j,k}{-}^{\left(h\right)}s_{i,j,k}^{\prime })}^{2}}$$

(8) $$|s_{i,j,k}-s_{i,j,k}^{\prime }|=\sqrt{\sum_{h=0}^{c-1}w_{h}\cdot {{(}^{\left(h\right)}s_{i,j,k}{-}^{\left(h\right)}s_{i,j,k}^{\prime })}^{2}}$$

If the variances and eventually the mean values of different sample components and/or streams *I* and $I^{\prime }$ differ, the generalized Formula (9) can be used.

(9) |si,j,k−si,j,k′|=∑h=0c−1wh·(si,j,k(h)−EhSDh−si,j,k′(h)−Eh′SDh′)2

Note that (7) and (8) are special cases of (9), which are usable when the differences $E_{h}-E_{h}^{\prime }$ and $SD_{h}-SD_{h}^{\prime }$ may be considered negligible.

Finally, if the variances and mean values are not known (or the computation takes too much time), the Equations (5) and (6) may be used separately for each attribute, resulting in (10) and (11), respectively. Note that the error thresholds $\varepsilon_{h}$ for different attributes may differ from each other.

(10) $${|}^{\left(h\right)}s_{i,j,k}{-}^{\left(h\right)}s_{i,j,k}^{\prime }|<\varepsilon_{h},0\leq i<ResX,0\leq j<ResY,0\leq k<ResZ,0\leq h<c$$

(11) $$\frac{1}{n}\sum_{i=0}^{n-1}{|}^{\left(h\right)}s_{i,j,k}{-}^{\left(h\right)}s_{i,j,k}^{\prime }|<\varepsilon_{h},0\leq i<ResX,0\leq j<ResY,0\leq k<ResZ,0\leq h<c$$

### 2.3. Data Restoration

The idea for digital data restoration comes from the physical restoration of works of art and architecture. Not surprisingly, it has made the strongest advances in the field of processing images with continuous colors (photographs), where a variety of well-established algorithms for growing (expanding) regions with soft color and shadow gradients, edge detection, segmentation etc., can provide quality reconstructions of missing or corrupted parts. The data restoration, also known as digital inpainting and initially also retouching, was first developed for the purposes of denoising, the removal of superimposed text like subtitles and dates, and the removal of entire objects from the image like microphones, wires, and aircraft trails. Bertalmio et al. [38] proposed a conceptually simple algorithm, which fills in the previously selected (detected) regions by completing the isophote lines arriving at the regions’ boundaries with information surrounding them. Based on this and other pioneering works, researchers quickly discovered the potential for the efficient compression of images and, gradually, other multimedia data. A survey was performed in [39]. The idea of this type of compression is to add noise or other changes and/or delete parts of the image (or other data streams) in a controlled way, so that the rest of it is more compressible by reducing the number of data and/or entropy, while the restoration process is still able to produce a decoded copy that is close enough in quality to the original. Wang et al. [40] proposed a structure-aware inpainting method to restore the skipped structural regions by taking advantage of the available portion of the decoded image. The method extracts a binary structure map to indicate the skipped regions and then propagates textures across this map. Liu et al. [41] proposed a conceptually similar method that divides the image into square blocks and analyses them. After that, some blocks are omitted, while the rest represent features that serve to restore the omitted blocks. Both methods are reported to be up to 50 % more efficient than JPEG.

However, the focus seems to have shifted (progressed in our opinion) to methods that do not propagate textures but are based on partial differential equations (PDEs) and variational techniques. Shen and Chan have based their research on the connectivity principle of human visual perception and presented a non-linear PDE inpainting model based on curvature-driven diffusion for untextured images, first on the second-order PDEs [42], then on the third-order ones [43]. At that time, the use of PDEs in image compression was limited to pre- and post-processing until Galič et al. extended it to the decoding step [44,45]. Their basic idea was to reduce the image data to a well-adapted set of meaningful sparse points that can be efficiently encoded. The decoding was performed by using these sparse data and interpolating them using edge-enhancing anisotropic diffusion (EED). The experimental results [46] showed that average errors by the R-EED (EED within a rectangular subdivision) codec [47] and the biharmonic tree codec [48] were mostly low compared to JPEG and JPEG 2000, especially at higher compression ratios. PDE-based image compression was later additionally improved by optimizing the selection of data (pixels) for inpainting [49,50] that yields the most accurate reconstruction. The process incorporates clustering and quantization built upon it, which correspond to the feature-selection step in the feature-based data compression. Outside of the PDE context, a hybrid method between vector quantization and data restoration was presented in [51]. The encoder divides the image into blocks in predetermined places and encodes them using vector quantization. Satpute and Tidke [52] combined a similar data compression approach, also based on side-match vector quantization and inpainting, with watermarking. A restoration-based method for audio compression can be found in [53].

The development of data compression algorithms based on digital restoration can also benefit from the knowledge of a related concept called compressed or compressive sensing [54]. This is a signal processing paradigm that allows the capture of fewer samples when the signal is sparse or compressible in a certain domain (e.g., frequency, wavelet). Donoho [54] revealed already in 2006 that most of the data acquired by our modern technology-driven civilization can be discarded with almost no perceptible loss. For example, music is usually stored and played back in compressed MP3 format, even though much more raw data were originally recorded. In compressive sensing, fewer samples are required to reconstruct sparse or compressible signals, which breaks through the traditional Nyquist–Shannon sampling theorem [55]. The paradigm immediately gained popularity in situations where sampling sources are limited by computing power and storage (edge computing, sensor networks, later IoT) or expensive (medical imaging). Data reconstruction algorithms perform intelligent upsampling, i.e., interpolation enriched by inpainting procedures. They are usually based on L1 norm (the sum of absolute values of errors) minimization, which can be very complex and time-consuming and consequently left to run on powerful network servers [56]. Gan [57] proposed block-compressed sensing for natural images. The proposed image reconstruction algorithm involves both linear and nonlinear operations such as Wiener filtering, projection onto the convex set, and hard thresholding in the transform domain. Stolojescu-Crişan and Isar [56] directly compared and connected the compressive sensing reconstruction with state-of-the-art inpainting algorithms. Li et al. provided an extensive survey of compressed sensing techniques [55]. Mishra et al. [58] addressed the energy consumption of data compression algorithms in wireless sensor networks. They proposed and analyzed a simple low-cost–energy combination of run-length encoding and adaptive Huffman encoding. Liu et al. [59] presented an adaptive sampling technique that enables sensor systems to intelligently prioritize and transmit the most relevant data. Their primary objective was to enhance the preservation of information that is critical for computer vision tasks where visual quality is usually not sufficient.

We may summarize that the existing data restoration methods are domain-dependent and lossy, while the utilized features are derived from artificial data structuring rather than from high-level relations. The examples below are not related to the described methodologies of texture mapping, PDE-based interpolation, vector quantization, or compressive sensing. They are intended to illustrate in a simple way how data restoration can be used in a lossless or at least near-lossless way.

Everyone is nowadays familiar with the ability of web browsers and text editors to autocomplete or autocorrect typed phrases. For example, if someone types in ‘UMIDID KIMDOM’, an ‘inteligent’ software will ask them if they might have wanted to type in ‘UNITED KINGDOM’. In this case, the two phrases are similar enough and the system ‘knows’ that the second one makes sense and the first probably does not. Of course, this similarity has its limits. If a user is more sloppy and types, e.g., ‘OMIDID KIMDOM’ or ‘UMIDID KIMDUM’, the system we used in this simple test did not associate these phrases with ‘UNITED KINGDOM’. And what does this bizarre example have to do with data compression and restoration? Let us assume that we input errors deliberately by substituting ‘T’ with ‘D’, ‘E’ with ‘I’, ‘N’ with ‘M’, and so on in a controlled way. By doing so, we increase the probability of some symbols and decrease the probabilities of others (in the extreme case, even omitting them from the alphabet), which generally has a positive effect on entropy and compression. In the idealized case where the phrase was isolated from the context (the alphabet consisted only of the letters of the phrase), we needed 43 bits (42.106) for ‘UNITED KINGDOM’ and only 29 bits (28.755) for ‘UMIDID KIMDOM’. Note that, besides the character pairs substitutions, we also omitted ‘G’ from ‘KINGDOM’. The example is analyzed in Table 1. Of course, the decoder must include a restoration capability, which in this case means a natural language processing algorithm with an associated dictionary.

Another example is from a continuous data domain and concerns the restoration of intermediate elements in some obvious sequence of values. For example, consider the sequence 〈1,2,3,4,5,6,7,8,9,10〉 (the commas are not part of the encoded stream). If we instead write 〈1,2,3,10〉 and apply the rule in the restoration algorithm that the apparent trend detected from a sub-sequence of the values is followed up to the next encoded value, we undoubtedly shorten the encoded stream considerably.

However, if the nature of the data is such that the sequence 〈1,2,3,10〉 can actually appear in the stream, we meet an ambiguity that can be resolved by an additional symbol. This tells the decoder whether or not to use restoration. For example, 〈1,2,3,…,10〉 should be restored into 〈1,2,3,4,5,6,7,8,9,10〉, while 〈1,2,3,10〉 is decoded as 〈1,2,3,10〉. The additional symbol ‘…’ must be sensibly chosen to minimize the increase in entropy and the compression ratio. Of course, in the ‘UMIDID KIMDOM’ example, we may also meet a similar ambiguity in particular contexts. Surely, each of us has encountered a situation where the proposed autocomplete and autocorrect functionalities were annoying. Here again, we can solve the problem by adding an extra symbol, or perhaps content ourselves with the near-lossless compression and then manual corrections of the decoded text.

To conclude, lossless restoration is generally an unsolvable problem, or the solution of possible ambiguities makes compression much less efficient than one would expect. Note that we should not explicitly indicate how many samples have been omitted or even indicate their positions in the stream, as this is then no longer omitting samples but a way of encoding them. For example, putting zeros at positions with obvious values typically translates to RLE, but explicitly indicating a large number of omitted locations may be even more wasteful than encoding sample values or residuals at the locations. However, even in practice, we encounter situations in particular domains where lossless restoration can be efficient. For example, when encoding voxelised waterproof surfaces with chain codes, even large areas of samples (pixel sides) on planar surfaces can be unambiguously omitted.

### 2.4. Data Compression and Machine Learning

The most recent progress in image, video, and audio compression was achieved by the incorporation of machine learning models, in particular neural networks. The latter combine the automated extraction of features with their use to predict signal values from their spatial or temporal context. They are also used to classify streams or their parts (blocks or segments) in order to select the most suitable data compression method or a repertoire of settings in hybrid approaches. Jiang et al. [60] introduced a technique of image compression that uses a pair of convolutional neural networks (CNNs). The first network generates a compact intermediate representation for further compression with a standard algorithm, while the second network improves the quality of the decoded image by deriving and applying the residual information about image details. A related procedure for digital audio compression was proposed in [61]. The prediction of residuals by CNN to perform lossless image compression was described in [62]. A similar approach is video compression with current block prediction from context features [63]. Toderici et al. [64] developed lossy image compression using recurrent neural networks (RNNs), which encode the differences between the original and the reconstruction in multiple iterations. Using this approach, the authors improved the results in comparison to JPEG with respect to perceptual metrics, which consider the sensitivity of human vision to certain distortions in the reconstruction. Compression with generative adversarial networks (GANs) is based on the generation of extremely compact code, from which the high-frequency part of the content is reconstructed [65,66]. The use of GANs for image compression is part of the broader field of generative visual compression [67], which leverages other deep generative models, such as variational autoencoders (VAEs) and diffusion models. A generative model is trained to learn compact latent feature representations based on the observed patterns in the data and reconstruct the original data from these latent codes. In a study by Liu et al., an autoencoder was used for the lossy compression of big data generated by scientific simulations on a high-performance computing system [68]. The results demonstrated multifold improvement of compression ratios in comparison with traditional lossy compressors used for floating-point data in scientific computing. Although the new concept shows great compression potential, it remains challenging to achieve robust reconstructions and efficiency outside of limited domains (e.g., images of faces) [69].

Deep learning was also used to improve individual steps of the HEVC method for video compression [70,71]. Features extracted by deep learning models are, to some extent, transferable between datasets within the same domain, but model fine-tuning is required in order to optimize the efficiency. The downside of these approaches is that their operation is not easy to interpret. The machine learning of such models is, in general, also controlled by a loss function that evaluates the reconstruction quality globally and is, therefore, less suitable for near-lossless compression.

### 2.5. Domain-Independent Data Compression

In practice, there are no efficient domain-independent data compression methods for geometric and/or multimedia data. General data compression programs such as ZIP, RAR, and ARJ compress data from different domains, but they only gain success with data sets from relatively small alphabets, combining traditional lossless statistical coding, dictionary compression, and RLE of repetitions. However, we do not know any lossy domain-independent (general) data compression algorithms, at least none that can be widely used in practice. There are a few lossy upgrades of the LZ77 algorithm. Yang and Kieffer [72] proposed two such general lossy data compression schemes, one with a fixed rate and the other with a fixed distortion. Luczak and Szpankowski proposed a similar method based on approximate pattern matching [73]. A version specialized for image compression was later derived [74] and achieved promising results comparable to JPEG, but unfortunately performed rather slowly. Of course, general lossless algorithms can pre-process the data stream, e.g., by quantization, truncation, or pruning, making the final results lossy, but this would not save much space compared to the lossless mode, except with unacceptably high losses. In more recent times, several lossy data compression algorithms have been specifically developed to run on high-performance computers. These algorithms include SZ [75], ZFP [76], and MGARD [77]. An extensive analysis was given in [78]. They are designed to compress structured multidimensional floating-point scientific data, which means that they can also process images, audio, video, or voxel grids. However, they are not optimized for them, as they do not explore e.g., psychoacoustic models such as MP3 or AAC or spatial redundancy and human visual perception such as different domain-specific lossy image compression algorithms. They are also less suitable for unstructured data such as point clouds or sparse voxel data. As a consequence, lossy domain-specific algorithms for individual multimedia data types or geometric models are advantageous compared to the general ones. Furthermore, features and restoration methods are also domain-dependent, although our methodology, COMPROMISE, presented in the next section will generalize them to some extent.

## 3. COMPROMISE

COMPROMISE (Data Compression Paradigm Based on Omitting Self-Evident Information) is a three-year basic research project funded from the budgets of the Republic of Slovenia and the Czech Republic. It is carried out by two complementary research teams of computer scientists from the University of Maribor and the University of West Bohemia in Pilsen. COMPROMISE aims to achieve the following objectives:

- To develop a general and universal data compression methodology with a unified taxonomy of features from diverse domains, and a common framework for lossless, near-lossless, and lossy compression.
- To upgrade the predictions of original data by integrating the techniques of feature selection and data restoration.
- To improve the compression ratios in lossless and near-lossless modes in comparison with the existing approaches.
- To improve the accessibility and reusability of features. Access to semantic features is often easier from a compressed stream than from raw data and feature-based restoration.
- To verify the paradigm in four pilot domains: raster images, digital audio, biomedical signals, and sparse voxel grids. During the project, the research has been extended to complete voxel grids, point clouds, and triangulated surfaces (static, dynamic, and time-varying). COMPROMISE’s results to date have been published in the following journal articles: refs. [13,79] on general issues (entropy coding and feature selection, respectively), refs. [80,81,82] on images, and ref. [83] on meshes.

The overall goal of COMPROMISE is thus to develop a universal and general data compression paradigm based on feature-based predictions and data restoration. This addresses the four main trends from Section 2. Universality means that the paradigm addresses lossy, near-lossless, and lossless compression, and generality means that the paradigm is largely domain-independent, providing a unified taxonomy of generalized features from diverse domains. The aforementined overall objective is further elaborated in the first two specific objectives above. In any case, we also want the solutions developed according to the proposed methodology to also be effective in terms of the compression ratio, as given in the third specific objective. The last two objectives relate to improved accessibility and reusability, validation, and dissemination. Note that COMPROMISE is not a strictly specified and implemented computer program but a methodological concept, an umbrella that integrates many existing domain-dependent and independent methods, supports hybrid lossless–lossy techniques, and encourages the development of new algorithms that can be highly competitive with state-of-the-art methods.

### 3.1. COMPROMISE Concept

**Figure 1:**
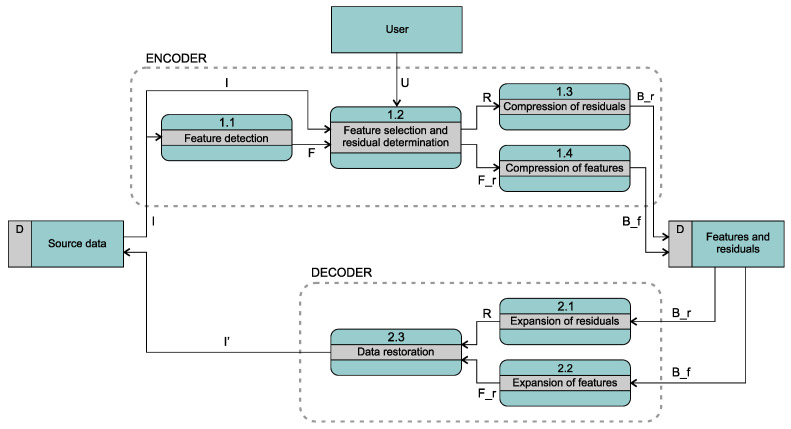
The COMPROMISE feature-based data compression encoding/decoding concept.

The overall goal and the specific objectives of COMPROMISE are achieved with the concept from Figure 1, where a data flow diagram of the COMPROMISE encoder and decoder is shown. It is a generalized COMPROMISE architecture that will be common to all COMPROMISE codec implementations in the pilot domains and possibly beyond. The encoder accepts the input data stream *I* and detects its features *F* (process 1.1). In the next module (1.2), the set of detected features is reduced through the optimization of the output data size and restoration abilities. The user-defined compression mode and allowed tolerances are also considered within this module (data flow *U*). This iterative optimization process also incorporates the data restoration method (the same method is used by the decoder). The described module 1.2 outputs two data streams: the reduced set of features $F_{r}$ and the residuals *R*. The latter contains the differences between the data in the input stream *I* and the most recently restored data generated within the module. The streams F_r and *R* are then encoded by a lossless data compression method (processes 1.3 and 1.4—two different methods may be used for the two streams). The resulting bit streams B_r and B_f are then stored in the file. The decoding of the compressed data in the bottom part of Figure 1 is significantly simpler. The decompressed data streams F_r (process 2.2) and *R* (process 2.1) are used to restore the data stream I’ (process 2.3). *I* and I’ are identical in the lossless mode but usually slightly different in the near-lossless or lossy mode.

The input and output data streams *I* and I’ and modules for feature detection (1.1) and data restoration (2.3) are domain-dependent (in part also the module 1.2 for feature selection and residual determination), and controlled by the domain identifier, which is transmitted with the data streams *I*, F_r, and B_f, while the coding mode information (*U*) is not needed by the decoder.

Feature extraction may be considered lossy compression, as its results *F* enable the reconstruction of a lossy data stream, i.e., decoding (expanding) features into a lossy sequence of samples. This lossy stream $I^{*}$ is actually a stream of predictions of all the samples from *I*. Its computation is hidden in modules 1.2 and 2.3 of Figure 1, but obviously, $I^{*}=I-R$ in the encoder or $I^{*}=I’-R$ in the decoder.

**Figure 2:**
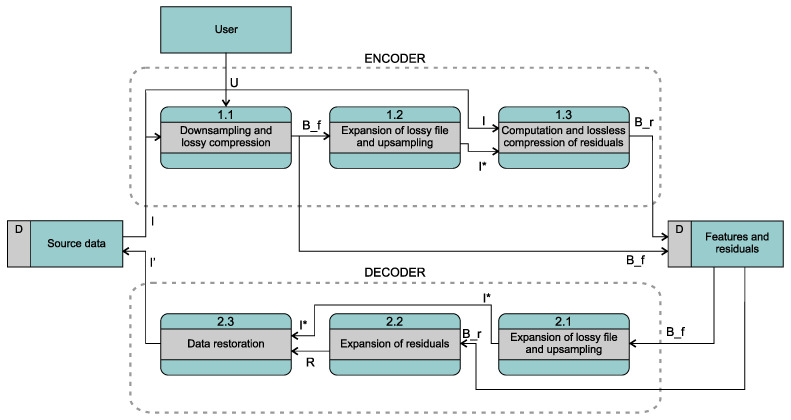
A variant of the COMPROMISE concept with lossy compression replacing feature extraction.

The observation that the decoded *F* is actually a lossy presentation of the sample predictions leads us to a simple variant of the COMPROMISE concept, shown in Figure 2. Instead of using the parsed feature representation as *F*, we use the output of an efficient lossy compression algorithm. Decoding this output then yields predictions $I^{*}$ which, together with the input set *I*, are used to compute the residuals *R*. We will analyze the concept using the example of digital audio in Section 3.3. Since the bit length of the compressed feature set B_f can still be quite large and the length of B_r is typically even larger, we extend the pipeline by downsampling (block 1.1) before lossy coding and upsampling after decoding the lossy feature file. Note that the latter need not be exactly the inverse process of the former, the only constraint is the same resolution of $I^{*}$ and *I*. In fact, it is even advisable to use some kind of intelligent upsampling technique, similar to that in the compressive sensing reconstruction. In any case, blocks 1.2 and 2.1 in Figure 2, which provide the expansion of the lossy file and upsampling in the encoder and decoder, respectively, should be identical.

**Figure 3:**
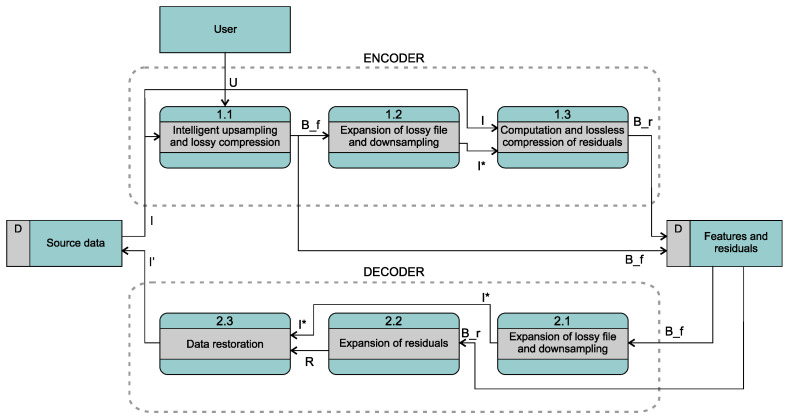
A draft of a compressive-sensing variant of the COMPROMISE concept.

A similar idea, but first performing upsampling and then downsampling after decoding, is presented in Figure 3. Here, it is mandatory to use intelligent upsampling in step 1.1, based on the compressive sensing and data restoration paradigms. The idea is to restore missing and/or corrupted samples as best as possible. The concept stems from the question of where we have higher-quality audio: if we losslessly compress a 44.1 kHz channel with 16-bit samples, or if we lossy compress a 192 kHz channel with 24-bit samples. The assumption is that perhaps by upgrading from lower to higher quality, we produce such better residuals that the higher resolution of the lossy part is worthwhile. However, we have not yet tested the idea in practice.

### 3.2. Unified Taxonomy of Generalized Features

A feature is a piece of information that possesses high discriminative/predictive value for human interpretation or machine processing. In COMPROMISE, we consider as a feature a pattern of samples, e.g., a segment in a segmentation. It is usually expected that the samples in a feature share some common property, which can be represented more compactly in comparison to the list of the incorporated sample values. A sample is an individual data item in a raw data stream. The sample is, for example, a discrete point on a line in 1D, a pixel in 2D, or a voxel in 3D. It is specified by the location and value. The former identifies the sample within the stream uniquely, e.g., a triple of indices (i,j,k) in (2) and (3). It can be given explicitly for each individual sample or implicitly through a topology, established by a uniform or hierarchical subdivision of the stream.

The sample value (a data attribute, e.g., the amplitude or height) is a sequence of bits at the sample location, structured according to the data type specification.

- Integers in the positional notation are highly convenient for compression. If transformed to non-negative values, residuals with multiple leading zeros, which may be simply omitted, are often obtained.
- Floating point numbers in the positional notation can be considered in the same way as integers. However, the scientific notation, e.g., IEEE 754, appears to be slightly more complex. Here, the replacement of an input value by a residual potentially lowers the exponent and shortens the mantissa by shifting its non-zero digits to the left.
- Multiple attributes, e.g., RGB color components or a pair of stereo audio samples, may also be attached to a single data stream sample or, alternatively, streams of individual components may be considered as separate data streams.
- Samples without values are also possible. This makes sense if we are interested in a geometric shape only, i.e., the samples that do represent the region of interest. This corresponds to the complete-grid data stream of Boolean values 0 and 1, where the non-shape samples (e.g., 0-values) are omitted.

Non-numerical sample values are currently not considered in COMPROMISE because of the inability to simply derive a residual as the difference between the input value (symbol) and its prediction. Although the symbols can be numerically encoded, e.g., by ASCII codes, it is hard to judge whether, for example, ‘B’ is better predicted by ‘A’ or ‘C’ than by ‘V’ or ‘W’. The residuals should thus be designed on other principles, e.g., probabilities of good and bad guesses, which are, however, hardly compatible with the COMPROMISE paradigm. Furthermore, text, DNA, and other symbol-based sequences usually require lossless compression only, which deviates from the idea of a universal data compression methodology.

Features detected from the input stream *I* represent the stream of domain-dependent features *F*. In module 1.2 of Figure 1, *F* is iteratively transformed into the reduced stream of features F_r through the optimization process of feature selection and simultaneous residual determination. The feature selection is performed by our own suboptimal dynamic programming filtering method [79], where the edges of the feature graph represent total bit lengths of features between the samples represented by the nodes, and the nodes store cumulative lengths from the beginning of the stream to the considered sample. The differences between *F* and F_r define a unified taxonomy of generalized features. These differences are as follows.

- Features in *F* may overlap, while the features in F_r do not.
- Each sample s∈I is addressed by exactly one feature.
- Domain-dependent patterns of samples in f∈F may be interrupted by gaps, and the patterns in F_r and the corresponding patterns of residuals in *R* represent connected intervals.
- Samples in f∈F may be arranged into 1D, 2D, or 3D connected or sparse structures, while those in F_r and *R* are always arranged into 1D intervals (lists, segments, or substreams).

The description of a generalized feature *f* from $F_{r}$ consists of the following.

- *f.interpretation*: definitions of the presence and structure of data in the other two components. These are actually instructions for the data restoration module 2.3 of the decoder, enabling it to develop linear streams of features and residuals into the corresponding 1D, 2D, or 3D structures of the restored output stream $I^{\prime }$. Furthermore, f.interpretation also stores the information on whether f.pattern represents the feature with its boundary, the interior, key samples, or some combination of these. Finally, in the lossy and near-lossless mode, the information on entering the losses must be defined, i.e., the quantization and/or pattern subsampling parameters.
- f.pattern: the sequence of samples from *I* affected by the feature. These samples might be coded directly in f.pattern, in the corresponding part of the residuals’ stream *R*, or as a combination, where the former contains the feature control samples and the latter encodes the remaining ones.
- f.prediction: unambiguous rules, which determine how the feature affects samples from f.pattern.
  - The interpolation function interpolates key samples and/or values on the segment border/box. The goal is to find such an interpolation that the residuals of the estimated sample values are ‘optimal’ (with the lowest entropy already or best compressible to achieve the lowest entropy).
  - The approximation function approximates samples of a given segment in an “optimal” way (best-fitting curve/surface). Key samples and or the segment border/box are used to define the control points to be fitted.
  - The extrapolation function predicts values of the observed pattern by using the values from some predefined neighboring pattern.
  For simplicity, we will survey some of these functions in the audio example in Section 3.3, where the interpretation is the simplest due to the 1D nature of the signal.

### 3.3. Examples of Feature Interpretation in Digital Audio Compression

**Figure 4:**
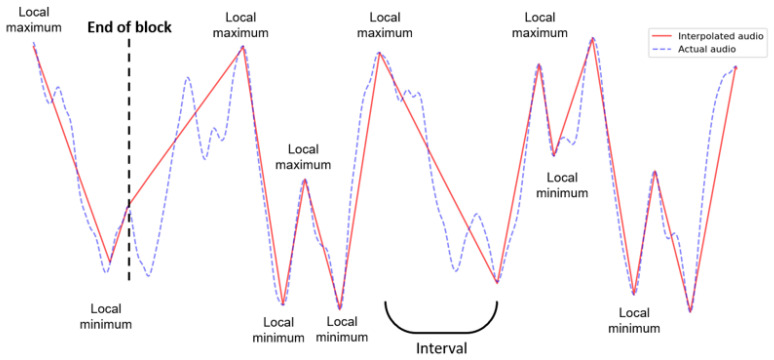
Interval features with linear approximation between two successive distinct extremes in a 1D (digital audio) example.

**Figure 5:**
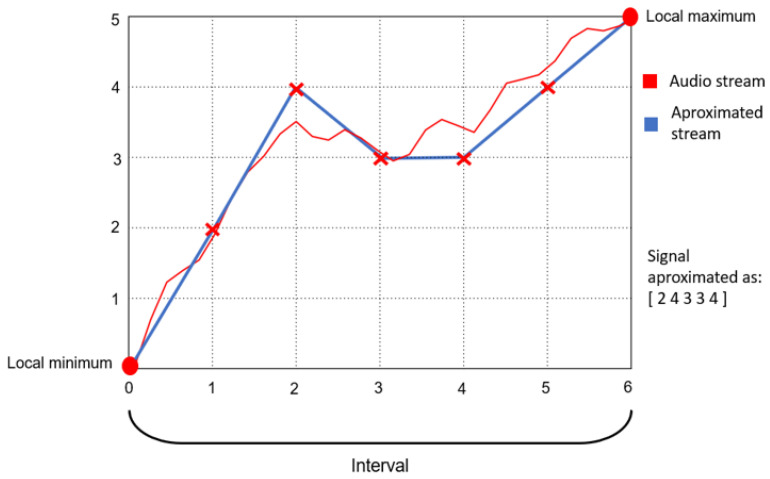
An interval feature with the grid-based mask polyline approximation between two successive distinct extremes in a 1D (digital audio) example.

Seven different prediction functions are used for the feature-based digital audio compression in the COMPROMISE methodology. All these functions are also applicable to other multimedia data types, either complete or sparse grids in 1D, 2D, or 3D. On the other hand, some interpolations or approximations, such as radial basis functions [84], are currently not used with audio yet. Feature detection in audio streams uses extreme-based features. In the input stream *I*, pairs of consecutive minimum and maximum values or vice versa are found, representing the feature interval borders. Within each interval, the following predictions are possible:

- Linear interpolation—the interval values are predicted with the line segment between the interval border values (see Figure 4).
- Average value approximation—all the values in the interval are predicted with the average value computed on the entire interval.
- The grid-based polyline interpolation (Figure 5) is based on a so-called mask, a predefined pattern of a few sample locations in a considered interval, aiming to locally attract the graph of the considered prediction function. At each mask location, a limited repertoire of sample values is offered, and the closest to the concrete sample value is chosen for the calculation of the residual. As there is a low number of mask locations and also a low number of predefined values at these locations, the mask can be compactly represented by only a few bits.
- Verbatim—lossless mode, where the interval values are explicitly listed.
- RLE—lossless mode, where the repeated value and the number of repetitions are given.
- Black-box-feature prediction—this is the implementation of a modified concept from (Figure 2, where a lossy compressed file replaces the feature set F_r.
- Data restoration—when an obvious trend exists in a sequence of the interval values, then the feature may be represented with a few initial values and the last value, while the missing intermediate values can be restored. The example 〈1,2,3,…,10〉 is explained in Section 2.3.

In Table 2, we compare the compression rate of digital audio in COMPROMISE lossless mode with the compression rate of the corresponding APE file generated with a popular Monkey’s Audio lossless compression software. The version 10.22 from September 2023 was used (there is no difference in compression ratio between it and the latest version 10.81 from November 2024). We initially experimented with FLAC as the most widely used lossless audio codec, but it turns out that Monkey’s Audio, when set to an Insane compression rate, typically produces a slightly smaller APE file compared to a FLAC file at the best compression rate. Of course, we felt it was fair to compare ourselves with the better of the two. In Row 1, we tested 50 files of different music genres and different lengths (full songs and shorter excerpts). We used the Linear, Average, Mask, Verbatim, and RLE feature classes, choosing for each interval the feature that gives the best compression. The compression ratio CR is the quotient between the original and the compressed file size, so higher values mean better compression.

**Figure 6:**
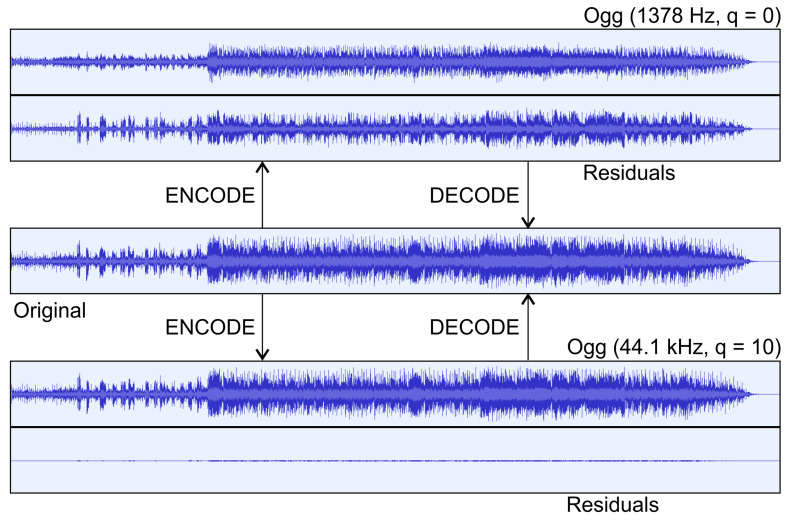
Compressing digital audio with the COMPROMISE variant of replacing feature extraction with lossy compression.

Features are not too space-consuming compared to residuals, so just a compact representation of each feature attribute was used, without any lossless entropy coding. On the other hand, binary adaptive sequential coding (BASC), Rice codes, Golomb–Rice codes, and interpolative coding were employed for residuals, and the one with the best compression was chosen in each case (which is not completely fair). In the last column, we show the difference between the APE-compressed original and the same input compressed with COMPROMISE. Here, we appreciate smaller values. In Row 1, this difference was between 0.08 and 0.16, which we are not happy with. We then conducted an experiment to see if it was even possible to beat Monkey’s Audio. We chose a strategy from Figure 2, where we used lossy audio compression instead of extracting and encoding individual features. Our first tests were based on MP3. However, this usually adds a shorter silence interval to the beginning when encoding, so the synchronization between the input file and the decoded MP3 file, which was needed for residual computation, was lost. Furthermore, the added silence is not always of the same length or at the same position. At the same time, MP3 only allows quality control via bitrate (96 to 320 kbps), so we opted for the fully competitive Ogg Vorbis instead. The latter does not add silence, and the quality can be controlled with resampling and with the quality parameter (0 to 10). In Row 2, we tried different settings of a selected rock track and obtained a difference between CRs in the rank of 0.01 and 0.14, much better than in Row 1. Row 3 refers to the lower part of Figure 6, where we have lossy OGG compression keeping the original (best) settings. The compressed original in APE format requires 19,057 KB, the lossy OGG file 5839 KB, the residuals in APE format 14,250 KB, and the sum of the latter two is 20,089 KB, which is converted to the CR difference results of 0.07. Row 4 refers to the upper part of Figure 6, where the image was heavily downsampled before OGG compression. As a result, the OGG file was only 313 KB, but the residual file was almost 5 MB larger than in the previous example. Row 5 shows an example where COMPROMISE achieved slightly better compression (only −0.01 difference, but still a success), which gives us additional motivation to further develop solutions not only for audio but also for other pilot types of multimedia and geometric data using the COMPROMISE methodology.

## 4. Discussion and Conclusions

The aim of this paper is to systematically identify the current challenges in data compression and the responses of the research community so far to highlight the problems and, where possible, to promote solutions. The focus is not on technological, environmental, economic, ethical, or security issues such as optimizing energy consumption, minimizing data acquisition and transmission in advanced networks using compressed sensing techniques, or performing complex enhancements of received or generated scientific data on supercomputers. Instead, our test paradigm COMPROMISE focuses on pure algorithmic innovations, introducing new predictive functions, transformations, optimizations, multimodality at higher semantic levels, reusability, universality, generality, etc. Recent trends driven by these algorithmic challenges are discussed in this paper. They include feature-based data compression, feature-related predictions, universal data compression that seeks to exploit the benefits of lossless compression in a lossy mode and vice versa, general data compression that leverages advances in different domains to develop efficient (almost) domain-independent solutions, data restoration and related intelligent compressive sensing reconstruction, and the use of machine learning in data compression. All these trends are highly interdependent and require a holistic approach to integrate them into new paradigms. For example, domain independence becomes problematic if one wants to use higher-level semantic features. A unified taxonomy and also a universal representation of generated features is then needed. This was addressed in COMPROMISE by means of a catalog of domain-independent features and by mapping 2D and 3D representations to 1D sets. The latter requires a small amount of domain-dependent data in each feature, aimed to perform the final mapping of decoded samples back to the original domain.

The implementation of the specific objectives of COMPROMISE from the beginning of Section 3 is not yet complete as the project is still ongoing. We have developed a unified taxonomy of features, but have not yet translated the domain-dependent features from all pilot domains into it. Similarly, we designed the concept of the integration of lossless, near-lossless, and lossy compression, but we have only recently started on the latter two. Even with lossless compression, we are not yet completely satisfied with the compression ratios of the data from all pilot domains. We can say that the inclusion of features and restoration are complete. Improved accessibility and reusability are ensured as the COMPROMISE concept is highly asymmetric and feature decoding is certainly significantly faster than feature detection and selection. We validated the applicability of the COMPROMISE paradigm in the audio domain, although research in the other pilot domains is at roughly the same level. We showed that the combination of state-of-the-art lossy and lossless algorithms can outperform the compression rate of a state-of-the-art lossless algorithm. In addition, we can optionally use the lossy algorithm alone without encoding the residuals. In general, COMPROMISE is a very comprehensive methodology in which a good number of existing compression algorithms can be accommodated. However, we would like to see our own implementation of a lossless algorithm, e.g., incremental coding, at least for residual compression, as soon as possible. Ultimately, we would also like to get rid of the use of OGG. We can still save the same space by performing entropy coding on features, which are only compactly written now. Besides this, we have not yet used the data restoration feature, which omits or deliberately corrupts individual samples. Perhaps the lossy OGG file could also be used in the same manner as all other feature types, i.e., on shorter segments. These do not have to be exactly the intervals we have mentioned but could also be longer blocks that would be encoded independently. Here, we will also need feature selection, where we can use the same algorithm [79] as for the interval features selection, but with a different optimization function, where we store the feature qualities in the graph nodes and correlations in the edges. However, once we have chosen the parameters that most affect the selection and compression settings, the feature selection can be extended to classify tracks and/or blocks by musical genre, enabling the potential use of an ensemble of different algorithms.

## Figures and Tables

**Table 1:** Digital restoration performed on “United Kingdom” use case.

Stream	Alphabet Size	Alphabet	# Of Symbols	Entropy	Min. Bitlength	Restoration Success
UNITED KINGDOM	10	D,E,G,I,K,M,N,O,T,U	13	3.239	42.106	Yes
UMIDID KIMDOM	6	D,I,K,M,O,U	12	2.396	28.755	Yes
UMIDID KIMDUM	5	D,I,K,M,U	12	2.230	26.755	No
OMIDID KIMDOM	5	D,I,K,M,O	12	2.230	26.755	No

**Table 2:** Comparison of compression ratios for different repertoires of features in audio data.

#	Genre	Features	OGG kHz	OGG Quality	Orig. APE CR	OGG + APE Res. CR	Comparison CR
1	Misc.	Linear, average, mask, verbatim, RLE	/	/	1.77 on average	/	0.08–0.16
2	Rock	Black Box	Misc.	Misc.	1.48	1.34–1.47	0.01–0.14
3	Rock	Black Box	44.1	10	1.41	1.34	0.07
4	Rock	Black Box	1.378	0	1.41	1.39	0.02
5	Classic	Black Box	2	1	3.48	3.49	−0.01

## Data Availability

The data presented in this study are available on request from the corresponding author.

## Abbreviations

The following abbreviations are used in this manuscript:

AAC	Advanced Audio Coding
ALS	Audio Lossless Coding
APE	Monkey’s Audio lossless file format
ARJ	Archived by Robert Jung
ASCII	American Standard Code for Information Interchange
BASC	Binary Adaptive Sequential Coding
CNN	Convolutional Neural Network
COMPROMISE	Data Compression Paradigm Based on Omitting Self-Evident Information
CPU	Central Processing Unit
DCT	Discrete Cosine Transform
DEFLATE	lossless data compression combination of LZ77 and Huffman coding
DNA	Deoxyribonucleic acid
ECG	Electrocardiography
EED	Edge-enhancing anisotropic diffusion
EEG	Electroencephalography
FLAC	Free Lossless Audio Codec
GAN	Fenerative Adversarial Network
HEVC	High Efficiency Video Coding
HPC	High-Performance Computing
ICT	Information-Communication Technology
IEEE	Institute of Electrical and Electronics Engineers
IoT	Internet of Things
JPEG	Joint Photographic Experts Group and their lossy image compression
JPEG 2000	JPEG and their wavelet-based image compression algorithm
JPEG-LS	JPEG Lossless image compression algorithm
LZ77	Lempel–Ziv universal lossless data compression algorithm from 1977
LZW	Lempel–Ziv–Welch universal lossless data compression algorithm
MDCT	Modified Discrete Cosine Transform
MGARD	MultiGrid Adaptive Reduction of Data
MGCC	Multiple Grid Chain Code
MP3	MPEG-1 Audio Layer III or MPEG-2 Audio Layer III
MPEG	Moving Picture Experts Group
MwI	Move with Interleaving transform
OGG	Ogg Vorbis lossy audio file format
PDE	Partial Differential Equation
PNG	Portable Network Graphics
RAR	Roshal Archive
R-EED	EED within a rectangular subdivision
RGB	Red, Green, Blue color model
RLE	Run-Length Encoding
RNN	Recurrent Neural Network
SD	Standard Deviation
SZ	Lossy compressor framework for scientific data
VAE	Variational Autoencoders
ZB	Zettabyte
ZFP	A very fast compressor and decompressor for floating-point data in HPC
ZIP	Universal lossless archive file format developed by Phil Katz
